# Regulatory Pathways of the Maturation-Related Decline in Adventitious Root Formation in Forest Tree Species

**DOI:** 10.3390/plants15132054

**Published:** 2026-07-02

**Authors:** Daniela Cordeiro, Alberto Pizarro, Carmen Díaz-Sala

**Affiliations:** Department of Life Sciences, University of Alcalá, 28805 Alcalá de Henares, Madrid, Spain; danielacordeiro@outlook.pt (D.C.); alberto.pizarro@uah.es (A.P.)

**Keywords:** adult cell reprogramming, organogenesis, polarity, propagation, regeneration

## Abstract

Vegetative propagation is widely used in forest plantations to propagate elite genotypes with traits of economic or ecological interest. However, the loss of the ability to form adventitious roots is a dramatic effect of tree age and maturation and represents a major limitation for the clonal propagation of high-quality genotypes. This review describes the evolution of our understanding—from the traditional role of plant growth regulators to current findings on cellular signaling—regarding the maturation-related decline of adventitious rooting in forest species. Evidence suggests that interactions between the cell wall, plasma membrane, and cytoskeleton play a key role in this process. Specific and dynamic modifications of the interactions between the cell wall and cytoskeleton could be possible targets for hormonal, developmental, environmental, and epigenetic regulation associated with the maturation-related decline in adventitious root formation.

## 1. Introduction

The recalcitrance of stem cuttings to adventitious root formation (AR) is a major limitation in the vegetative propagation of elite germplasms of many forest tree species, especially at the mature stage [1,2,3,4]. Adventitious rooting is a postembryonic organogenesis process of root induction from differentiated cells that have not been specified to develop a root, at positions where they do not normally occur during development. Adventitious roots can be induced in intact plants, or on cuttings, leaves, or roots of some species by a regulated process of cell proliferation and differentiation into roots [5,6]. The cellular events leading to adventitious rooting on cuttings have been reported in several gymnosperm and angiosperm forest species [7,8,9]. Root meristems can be induced directly on specific cells of cuttings or indirectly from the callus that forms at the wounding site [10,11]. Although many factors account for the induction of AR on cuttings [9], we will focus on the effect of maturation and age of the tree, which is a major limitation for the clonal propagation of high-quality genotypes. The maturation-related decline in AR has been studied in key forest species. Extensive research is well documented primarily in Pinaceae (pine), Fagaceae (chesnut and oak), Salicaceae (poplar), and Myrtaceae (*Eucalyptus*), although data have also been published on Juglandaceae (*Juglans*) and Aquifoliaceae (*Ilex*).

The recalcitrance of AR at the maturation stage is related to the requirement of cuttings for de novo organization of meristems [11]. Histological observations on the development of adventitious roots in hypocotyl cuttings, an organ of embryonic origin of conifer species, reveal that roots always form directly from a small region of the cortical parenchyma located centrifugally to the resin canal, which marks the poles of the primary xylem. The time course of events leading to AR is similar among distinct species in the presence of exogenous auxin, the main inducer of adventitious roots [11,12,13]. No cell divisions were observed during the first 2 days of auxin treatment; during this period, cell expansion and reorganization were the only cellular responses to auxin treatment in loblolly pine (*Pinus taeda* L.) [11], lodgepole pine (*Pinus contorta* Dougl. ex Loud) [12], or Eastern white pine (*Pinus strobus* L.) [13]. Mitosis in areas of future root meristems occurs between 2 and 6 days of auxin treatment; however, rapid cell divisions are not detected before 6–8 days, and organization of root meristems does not occur before 10–12 days. In some species, such as lodgepole pine and Eastern white pine, non-treated hypocotyl cuttings form root primordia at a lower frequency and more slowly than auxin-treated cuttings. In both cutting types, non- and auxin-treated, the origin of adventitious roots and cellular changes leading to root formation are similar [12,13]. In angiosperm species, like *Castanea* or *Quercus* species, cells in the cambium, neighboring areas, and other tissues, such as the ray parenchyma and phloem region, divide periclinal and anticlinal in response to the presence of exogenous auxin. Cells showing meristematic characteristics are observed after 3–4 days of auxin treatment [7,8,14]. Although with low frequency, adventitious roots can also be induced in rooting-competent chestnut (*Castanea sativa* Mill.) microshoots maintained in auxin-free media. Roots seem to originate from the stem directly after two months of culture [15]. Rooting capacity is delayed or absent in auxin-treated epicotyl and woody cuttings from young trees when compared with hypocotyl cuttings from many conifer species [10,11,13,14]. In most cases, when roots were formed, they were induced from callus tissue at the cutting base. Tracheid nests, in association with small local areas of meristematic cells, are the first visible signs of differentiation within the callus. In general, root initials form in or near active callus meristems, giving rise to root primordia [10,16]. In Eastern white pine, roots emerged directly from vascular tissues of auxin-treated epicotyl cuttings from 11-week-old seedlings, with a similar pattern of development shown in hypocotyl cuttings; however, the timing and frequency of root primordium formation were delayed [13]. In non-treated epicotyl cuttings, roots were induced from callus tissues in a very small number of cuttings [13]. In loblolly pine, auxin-treated epicotyl cuttings from 50-day-old seedlings rooted poorly or did not root at all [11]. Cambial and cortical cell expansion were evident in epicotyl cuttings within the first 5 days of exposure to auxin, like the results obtained using hypocotyl cuttings; however, subsequent events leading to root regeneration occurred much more slowly and infrequently in epicotyl cuttings, while no callus was induced. The base of an epicotyl cutting from a young pine seedling consists mostly of primary tissue, although the cambium is already developed, with interruptions only at the primary leaf-axillary bud traces, and a complete ring of secondary xylem is differentiated [11]. Epicotyl cambial cells respond to the presence of exogenous auxin by dividing, but the reorientation of division planes needed for the de novo organization of a root meristem does not occur or occurs infrequently [10,11,13,16]. Similarly, in rooting non-competent microshoots of chestnut, the progression of cell divisions initiated in the cambial derivatives, especially on the phloem side, failed to organize properly into a meristem but rather gave rise to a callus structure [7].

In summary, cellular changes associated with root induction are similar for gymnosperm and angiosperm forest species. The auxin response of epicotyl and woody cuttings from young trees with low or null rooting capacity indicates that cells respond to the presence of rooting stimuli, but only specific cells at specific developmental stages form an adventitious root meristem. Several interrelated pathways may be involved in the cell plasticity required for the regeneration of forest tree species [3]. The effects of plant growth regulators, the spatiotemporal cellular and tissue signals, the crosstalk between hormones and key transcriptional regulators, and a dynamic cascade of regulatory changes in gene expression involved in organ patterning, such as epigenetic regulation mechanisms, regulate the cell fate-related modifications that enable a differentiated somatic cell to reactivate programs that lead to the induction of adventitious root meristems [17,18,19,20]. This review describes the evolution of our understanding—from the traditional role of plant growth regulators to current findings on cellular signaling—regarding the maturation-related decline of AR in forest species. Most of these studies are based on the association between phenotype and the physiological-molecular data. Although new data on the process has been published in recent years, it is not yet possible to establish either unified molecular models or the signal transduction pathways that determine whether rooting occurs. However, data pave the way for elucidating the complete regulatory signaling pathways and for guiding future research and manipulation of the process.

## 2. Hormones and Maturation-Related Decline of Adventitious Root Formation

Auxin plays a key role in the onset of AR [21], with exogenous auxin or auxin conjugate treatments being an absolute requirement to induce AR in most tree species [4,22]. Indeed, rooting recalcitrance in difficult-to-root species of *Eucapylptus* has been associated with relatively lower cambium-auxin content as compared to easy-to-root species [23], and the modification of auxin concentration and sensitivity during early age-related decline in the rooting competence of young *Eucalyptus globulus* Labill. seedlings have been described [24]. Nevertheless, differences in adventitious rooting capacity among individual cuttings of Scots pine (*Pinus sylvestris* L.), between rooting-competent hypocotyl and non-competent epicotyl cuttings from different families of loblolly pine, and between in vitro cultured juvenile basal sprouts and mature crown shoots of *Castanea* or *Quercus* species, were not related to the content, uptake, or metabolism of auxin; therefore, auxin does not account for all the developmental or genetic variation in rooting ability [7,8,11,15,25]. In addition, the pronounced decline in the rooting potential of mature cuttings or mature tissue culture explants is not paralleled by differences in the rooting response to auxin [7,8,11,17,26]. The initial hypothesis that non-competent mature tissues would not respond, or would respond more slowly to auxin than competent tissues in terms of reorganization and cell division, was rejected in pine. Rooting-competent and non-competent cells of hypocotyl and epicotyl cuttings of loblolly pine and Monterey pine (*Pinus radiata* D. Don) respond similarly during the early stages of root induction; both types of cells re-enter the cell division cycle, but only cells at specific locations in hypocotyl cuttings form an adventitious root meristem [11,17,18,26,27,28]. Reorientation of cell division planes in response to exogenous auxin during cell plate formation is one of the most evident changes characterizing rooting-competent cells in gymnosperm and angiosperm tree species, as compared to the periclinal divisions of non-rooting cells, or with callus-forming divisions, induced by auxin in non-competent cuttings [7,11]. Greenwood and colleagues determined that the sequence and timing of cellular reorganization, the onset of cell division, and the mitotic frequency were the same in disks from rooting-competent hypocotyl cuttings and non-competent epicotyl cuttings [26]. In addition, auxin-induced gene expression of a member of group II of GRETCHEN HAGEN 3 (GH3) correlated with the maturation-related ability of mature microshoots of chestnut to form roots, suggesting not only a role for *CsGH3-1* in regulating auxin homeostasis associated with different developmental stages of rooting cells, but also the capacity of non-competent mature microshoots to respond to auxin [29]. Vielba and colleagues described that auxin-related signaling was induced in rooting-competent and non-competent microshoots; however, the specific response in each case was significantly different [30]. Ayala and colleagues described that *AUXIN RESPONSE FACTOR 6*, involved in indole-3-acetic acid (IAA) signaling, increased due to age effects, while *GH3-1*, involved in IAA conjugation, decreased in adult shoots, probably because of a higher free IAA content in the juvenile stage, in *Eucalyptus nitens* Deane & Maiden [31]. These results indicate that the decline in rooting ability at the mature stage does not seem to be due to a lack of auxin response, and may be associated with a loss of cells capable of fully responding to auxin for the induction of adventitious roots in distantly related forest species [7,8,11,15,17,18,26,29,30,31].

Other hormones, such as cytokinins, abscisic acid (ABA), salicylic acid (SA), jasmonic acid (JA), gibberellins, and ethylene have also been reported as involved in AR in forest tree species in response to many factors [28,30,31,32,33,34,35,36,37]. Regarding the induction of AR associated with maturation, recently, cytokinin, ethylene, ABA, JA and SA content and signaling have been involved in the maturation-related decline of AR in chestnut, as well as in the adventitious rooting competence of poplar genotypes [30,33,35,38]. Rooting rate was significantly improved in mature chestnut microshoots in response to chemicals that modify ethylene biosynthesis, such as 1-aminocyclopropane-1-carboxylate or aminoethoxyvinylglycine, or signaling, such as silver ions, before or during auxin treatment, suggesting that ethylene may negatively influence the induction of adventitious roots in mature microshoots showing low rooting responses. However, ethylene showed a limited effect in the rooting response of juvenile microshoots, with high rooting ability, suggesting that ethylene response depends on the ontogenetic state of plant material [38]. Gene expression analyses suggest that abscisic acid might also negatively influence rooting in mature microshoots of chestnut [30,38]. In addition, specific components of the cytokinin signaling pathway were upregulated at the base of the stems of adult shoots of *Eucalyptus globulus* and *E. nitens* [24]. In summary, the variation in auxin response is dependent on species, developmental stage, tissue type, and experimental conditions. Research shows that the maturation-related decline in AR is influenced not only by variations in the auxin content or sensitivity, but primarily by the decline of cell capacity to respond to auxin in terms of AR, and its interaction with other hormones, among others.

## 3. Polar Auxin Transport, Auxin Distribution, and Maturation-Related Decline of Adventitious Root Formation

The role of polar auxin transport (PAT) in the formation of adventitious roots is well known [9,11,32,39,40,41,42,43,44,45]. For instance, recently, the improvement of the rooting capacity of mature chestnut microshoots in the presence of chemicals that modify ethylene synthesis or signaling has been associated with the modification of the expression of different genes encoding auxin transport proteins [35,38]. In addition, the inhibition of AR by gibberellins in pine, chestnut, and poplar has also been linked to the modification of polar auxin transport [28,34,46]. Nitric oxide influences rooting, affecting auxin signaling, in *Eucalyptus grandis* W.Hill ex Maiden [47]. On the other hand, PAT inhibitors, such as 1-N naphthylphthalamic acid (NPA), inhibit the induction of AR in the presence of exogenous auxin of rooting-competent hypocotyl cuttings of young loblolly pine seedlings if applied during the first 3 days of treatment, but do not affect auxin concentration or metabolism at the rooting site [11]. When NPA was applied in the presence of exogenous auxin to rooting-competent chestnut microshoots, there was a significant decrease in the rooting rate and a delay in the emergence of the roots [15]. These results suggest a role for the endogenous hormone in the AR process, or that exogenous auxin should be loaded onto an appropriate transport system.

Although no differences in auxin uptake, transport, accumulation, or metabolism were found between rooting-competent and non-competent cuttings at the base of the cutting in distantly related forest tree species [11,15], an asymmetric auxin distribution was detected in rooting-competent tissues of hypocotyl cuttings of Monterey pine seedlings after excision, which was, at least, maintained during the initial 24 h of root induction [27]. Treatments with NPA, which inhibits rooting and does not change the number of cell layers in the vascular cylinder, cortex, or pith, inhibited the asymmetric auxin distribution and the formation of an auxin gradient in rooting-competent tissues [11,27]. Asymmetrical distribution was not observed in non-competent cuttings, resembling the pattern observed in rooting-competent cuttings treated with NPA [27]. Auxin signal was more diffuse and evenly distributed through all tissues. Failure of non-competent epicotyl cuttings to root does not seem to be linked to the decline of the rate of PAT within the cutting [4,11,48]; however, results of auxin distribution in non-competent epicotyl cuttings and in competent cuttings treated with NPA indicate that PAT might also be required for auxin flow, localization, and distribution at the tissue or cellular levels [11,27]. Therefore, rooting-competent tissues retain an intrinsic capacity to maintain or accumulate auxin after excision. This auxin gradient and the asymmetric auxin distribution in the rooting cells of competent hypocotyl cuttings could be a major driver of AR in pine, and the lack of auxin gradients in non-competent cuttings could be crucial for the maturation-related decline of AR [27].

Auxin distribution largely depends on the dynamic expression and subcellular localization of the PIN-FORMED (PIN) auxin carrier proteins [49]. However, PIN activity can be modulated by endogenous or exogenous signals, such as other hormones, stress, or tissue-specific factors, to trigger developmental decisions that could initiate regeneration by triggering cell fates or other local changes [50]. Indeed, the stress response associated with wounding has been related to de novo regeneration [51,52]. No differences in the wounding stress response were observed between rooting-competent and non-competent pine cuttings [53]; therefore, other tissue-dependent signals could trigger re-patterning either by inducing cell-fate re-specification or by re-establishing the auxin distribution. Diphenylurea derivatives enhance AR in Monterey pine hypocotyl cuttings, distantly related herbaceous and woody species in the presence of endogenous or exogenous auxin [54,55]. Diphenylurea derivatives modify the distribution of auxin, which is localized in globular-shaped structures of cell divisions located centrifugally to the resin canals, at the positions of adventitious root formation in Monterey pine [55].

The requirement for PAT in the induction of AR and the asymmetric distribution of auxin detected in rooting-competent tissues after excision and during the early stages of AR in pine [11,27] could indicate the involvement of proteins regulating PAT in AR. In Monterrey pine, the expression patterns of the genes encoding PAT proteins—which belong to the AUXIN1/LIKE-AUX1 (AUX/LAX) and PIN families, are grouped based on the presence of exogenous auxin rather than on the age and rooting capacity of the cuttings or the temporal progression of AR induction, both in cuttings with rooting capacity and in those without it during AR induction. In general, these genes were expressed primarily in an auxin-dependent manner during AR, with the responses of competent and non-competent cuttings being very similar [56]. Two major expression patterns were defined based on hierarchical clustering during AR: (1) Genes showing similar or downregulated expression levels in auxin-treated competent and non-competent cuttings at the early stages of AR. This cluster was mainly enriched with *AUXIN INFLUX TRANSPORTER PROTEIN 1 (AUX1)* and *PIN1*; and (2) Genes with higher expression levels in auxin-treated competent and non-competent cuttings during AR. This cluster was mainly enriched with *PIN2* and *PIN3*. Variations in mRNA levels in auxin-treated competent and non-competent cuttings could indicate variations in PAT, and, perhaps, the redundancy of different proteins during development. The downregulation of *AUX1* and *PIN1*, and the upregulation of *PIN2* and *PIN3* in the presence of auxin suggest that auxin may regulate its own transport in rooting-competent and non-competent cuttings and, perhaps, the auxin distribution and auxin maxima could be regulated by the expression levels of these genes and proteins during AR. However, the close responses between rooting-competent and non-competent cuttings to auxin suggest that not only variations in the gene expression patterns but also additional signaling pathways, perhaps involved in the distribution and polarity of auxin transport proteins in the membrane, could also be involved in the variation in the auxin distribution and the auxin maxima in rooting-competent cells compared with non-competent cells during AR in pine [27]. PIN proteins can move very dynamically and change their positions within cells and tissues. They continually cycle between the plasma membrane and endosomal compartments, increasing local auxin levels [57,58]. Therefore, auxin maxima at the rooting cells during AR may also be driven by the dynamic redistribution of auxin carriers in rooting-competent cuttings. Localized modifications in the amount, availability, distribution, or activity of polar auxin transport proteins in rooting-competent tissues and the possibility that the failure of rooting non-competent cuttings to root is linked to a lack of those modifications, or subsequent steps in the signal transduction pathway, should be considered.

Mechanical and physical forces, which lead to a modification in the alignment of the microtubules that mark the position and orientation of the new cell wall and division plane after mitosis, as well as PIN repolarization, are involved in morphogenesis [59]. For example, PIN1 distribution correlates with the principal direction of mechanical stress in the leaf primordium, and growth-induced mechanical stress in the boundary zone upregulates PIN1 accumulation [59]. The polar distribution of PIN auxin carriers is maintained by cell wall–plasma membrane–cytoskeleton interactions [60,61], among others, which could be involved in the regulation of auxin distribution, and, consequently, root induction [62]. Therefore, changes in the physical properties of the cell or tissue, resulting in the modification of cell polarity and the mechanical or physical aspects underlying modifications of cell division planes, could be involved in the regulation of the maturation-related decline of AR in pine [19,63].

## 4. Cell Wall–Plasma Membrane–Cytoskeleton Continuum and Maturation-Related Decline of Adventitious Root Formation

Cell wall integrity signaling is used by distantly related organisms to adapt the cell walls during growth and development [60,61]. The impairment of cell wall integrity affects plant developmental processes in response to mechanical modifications of cell walls, including cell cycle progression, cell expansion, and organogenesis [64]. Cell wall integrity signaling involves several components and pathways, including receptor-like kinases (RLKs), ion fluxes, RAC/ROP guanosine triphosphate (GTP)-binding proteins, or cytoskeleton remodeling proteins, which may control the auxin distribution [64,65,66]. The RLKs, including lectin-type RLKs (LecRLKs), leucine-rich repeat RLKs (LRR-RLKs), wall-associated kinases (WAKs), and *Catharanthus roseus* RLK1-like kinases (CrRLK1Ls), play significant roles in the signal transduction of cell-wall-related signals. However, other cell wall and plasma-membrane-localized proteins may also play roles in cell wall signaling, such as stretch-activated ion channels or arabinogalactan proteins [64,65,66]. Plant RAC/ROPGTP-binding proteins are membrane-associated proteins involved in many signaling processes, and specifically, their roles during polar growth are recognized as fundamental mechanisms involved in cell polarity [64,65,66]. Plant RAC/ROP GTP-binding proteins may also play roles in transducing cell-wall-related signals because specific members of this family act downstream of RLKs, especially CrRLK1Ls [64,65,66]. ROP signaling is also involved in the regulation of cytoskeleton reorganization, auxin carrier polarity, and auxin distribution [64,65,66]. A role for the interaction between the cell wall–plasma membrane–cytoskeleton has been proposed as being involved in the maturation-related decline of adventitious root formation in pine [17,18,19]. Specific and dynamic changes in the interactions between the cell wall and cytoskeleton, affecting the polarity of auxin efflux carriers in rooting progenitor cells prior to and after the irreversible decline of adventitious root formation, may represent possible targets for the developmental, environmental, hormonal, and epigenetic regulation of the maturation-related decline in adventitious root formation [17,18,19,28,56,63].

The role of the cell wall–plasma membrane–cytoskeleton *continuum* in the maturation-related decline in AR has been thoroughly analyzed in Monterey pine [56]. The cambium cells exhibited active multiplicative divisions to maintain the periclinal orientation of the division plane in cuttings with and without rooting ability. However, in cuttings showing rooting ability, rapid and active formative divisions were observed in the cambium cells linked to the resin canals, associated with a change in the orientation of the division plane, which led to the organization of an adventitious root meristem [56].

Expression of genes encoding cell wall integrity sensors, and the signal transduction modules involving small guanosine triphosphate (GTP)-binding and cytoskeleton-associated proteins, has been associated with the capacity to form adventitious roots prior to the resumption of cell division and during the rapid cell division that leads to the organization of an adventitious root meristem [56]. A comprehensive analysis of the response of the cell wall, plasma membrane, and cytoskeleton in cuttings with and without rooting ability shows that gene expression profiles cluster based on the age and rooting ability of the cuttings, rather than on auxin treatment or the timing of induction during AR formation. Therefore, the coordination of the cell wall–plasma membrane–cytoskeleton response, which is involved in cell wall modification, microtubule organization, membrane trafficking, and, perhaps, auxin transport and distribution, may regulate cell behavior to respond to auxin and to induce an AR program [56].

Differentially expressed cell wall genes, in rooting-competent cuttings and non-competent cuttings of Monterey pine, at the time of excision and during AR, are functionally associated with pectin and hemicellulose metabolism, indicating a potential role of cell wall remodeling in the growth and maturation-related decline of AR in pine. These genes are mainly expressed in an organ- and age-dependent manner during AR in response to wounding and auxin. Cell wall remodeling is both an early response, before resuming cell division, and a late response, after the induction of rapid cell division, to wounding and auxin in both competent and non-competent cuttings under conditions of AR induction. Differential expression of key components involved in cell wall loosening and remodeling, such as expansins, specific pectin and polygalacturonase isoforms, and both xyloglucan- and pectin-modifying enzymes, may indicate a requirement for modifications in cell wall softening/stiffening to modify or maintain cell wall integrity depending on the cutting, age, or presence of auxin [48,56,62,67,68,69,70,71,72]. Modifications of cell wall integrity may be directly or indirectly relevant for AR in competent cuttings [56]. However, the close responses between competent and non-competent cuttings at specific times suggest that additional signaling pathways may be involved in the regulation of adventitious rooting capacity at the mature stage of development [56].

Perturbation of cell wall integrity reveals sensing mechanisms to transduce cell wall impairment signals and maintain developmental homeostasis. Genes encoding receptor-like kinases (RLKs), belonging to the lectin-type RLKs, wall-associated kinases, *Catharanthus roseus* (L.) G.Don. RLK1-like kinases (CrRLKs), and leucine-rich repeat RLKs, and mechanosensitive channels are mainly expressed in an organ- and age-dependent manner during AR in response to wounding and auxin, showing a major association with the maturation-related decline of AR. Gene expression of most *RLKs* analyzed seems to be downregulated at the time of excision and during AR for root induction in rooting-competent cuttings [56], perhaps to avoid specific functional redundancies and compensation involved in the maintenance of tissue and organ integrity [73]. The hypothesis that downregulation of RLKs may help maintain the competence to root by loosening the cell wall–plasma membrane signaling adhesion should be considered. Specific members of the WALL-ASSOCIATED KINASES (WAK) family physically interact with the wall [74]. Interestingly, this system is involved in cambial activity and xylem development in poplar [75,76]. The upregulation of *WAK2* and *LEUCINE-RICH REPEAT-RLK FEI* in response to auxin indicates that specific members of the *RLK* gene family may positively regulate cell behavior in response to cell wall perturbations.

Downstream regulators of the small GTP-binding protein family are also correlated with the maturation-related decline of AR. Small GTP-binding proteins play pivotal roles in diverse cellular processes, such as RLK signal transduction, cytoskeletal organization, cell polarity, cell proliferation and differentiation, intracellular membrane trafficking, transport vesicle formation, and nucleocytoplasmic transport [77]. Differentially expressed genes in rooting-competent and non-competent cuttings at the time of excision and during AR are functionally associated with RAN-, RAB-, and RHO-small GTP-binding proteins, indicating potential functions in endomembrane trafficking, RLK signal transduction, and cytoskeleton dynamics, among others, in the growth and maturation-related decline of AR in pine [56]. These genes are expressed in an organ- and age-dependent manner during AR in response to wounding and auxin, suggesting that these genes may be directly or indirectly related to the decrease in AR associated with maturation. The downregulation of specific *RHO*- and *RAN-GTPases*, as well as specific *RHO* regulatory factors involved in the deactivation of GTPases, occurred in competent cuttings. The differential expression levels of these genes may be related to the downregulation of *RLKs* in the same cuttings caused by wounding and auxin treatments, because RAC/ROP GTPases have been described as downstream signaling elements of specific CrRLK1Ls [78,79]. The upregulation of *RAB-GTPases* and specific *RHO* regulatory factors, involved in the activation of GTPases in competent cuttings during AR, may reflect the specificity of GTPases in the responses of various tissues at different developmental stages. Members of the ADP-ribosylation factor GTPases, such as GNOM and ARF-GAP proteins, are involved in AR [80,81]. Perhaps all genes have regulatory roles in the maturation-related decline in AR in response to wounding and auxin, as well as in modifications of the cell wall and RLK signal transduction, but in diverse developmental and environmental contexts and tissues, because each member of the GTPase gene family has its own unique expression pattern under wounding and auxin treatments.

A specific member of the *Arabidopsis* RAB GTPases, RAB-A5c, controls the growth direction during lateral root formation and organogenesis, interacting with cortical microtubules and cell wall mechanical properties [82,83]. Cytoskeleton-related genes are expressed in an auxin-, age-, or development-dependent manner in rooting-competent and non-competent stem cuttings during AR. Indeed, the cytoskeleton seems to play a role in adventitious rooting [84], and microtubules and microtubule-associated proteins are involved in AR in several species [85,86,87,88]. Differentially expressed genes, in rooting-competent and non-competent cuttings at the time of excision and during AR, are functionally associated with microtubule-associated proteins, indicating potential roles for microtubule dynamics in the growth and maturation-related decline of AR in pine. Although the initial gene expression in response to auxin is closely related in both competent and non-competent cuttings, the differential gene expression at late stages indicates that the differential dynamics of microtubules may be related to the maturation-related decline in AR after the induction of rapid cell divisions, resulting in meristem organization. Similar results have been described by Abu-Abied and colleagues in *Eucalyptus grandis* [85]. A fine-tuned crosstalk between microtubules, cell walls, and auxin transport is also required for proper adventitious root induction in *Arabidopsis* [86,87]. This crosstalk could be directly or indirectly relevant to AR in rooting-competent cuttings in response to wounding and auxin. The low mRNA levels of key components involved in the stabilization of microtubules and microfilaments, such as SCAR3, AUXIN INDUCED IN ROOT CULTURES9 (AIR9), and SPIRAL2/TORTIFOLIA1 (SPR2/TOR1), or the high mRNA levels of proteins involved in the organization and orientation of the cytoskeleton, such as AURORA kinases, KINESIN, and the MICROTUBULE-ASSOCIATED PROTEIN MAP65, in competent cuttings at late stages of the process, may be related to the flexibility of the cytoskeleton needed to organize an adventitious root meristem [56]. The different expression patterns of different family members in rooting-competent and non-competent cuttings during AR reflect the specificity of the responses in the various tissues and/or developmental stages.

Overall, these results indicate that genes encoding proteins of the cell wall–plasma membrane–cytoskeleton *continuum* are mainly expressed in an organ- and age-dependent manner in rooting-competent and non-competent stem cuttings of Monterey pine at the time of excision and in response to adventitious root induction. Also, the results suggest roles for the regulatory systems involving sensors of cell wall and membrane disturbances and downstream regulators belonging to the small GTP-binding family in the maturation-related decline of AR. Their functions may be associated with specific modifications of cell wall and cytoskeleton gene expression dynamics [56].

An array of master transcription factors may directly or indirectly control cell wall integrity and thus generate domains with specific growth parameters and identity. In addition, plasma membrane–cell wall adhesion signaling processes may lead the pathway to achieve specialized function and their reprogramming via induction or repression of specific transcriptional networks.

## 5. Potential Downstream and Upstream Signaling Pathways Associated with the Cell Wall–Plasma Membrane–Cytoskeleton Continuum and the Maturation-Related Decline of Adventitious Root Formation

Several transcription factors have been identified for their role in AR in tree species [43,63,89,90,91,92]; however, how their expression shifts in response to maturation is not properly characterized for most of them. Sánchez and colleagues and Solé and colleagues described a *SCARECROW-LIKE* gene in Monterey pine (*PrSCL1*) and in chestnut (*CsSCL1*) and a *SHORT-ROOT* gene in Monterey pine (*PrSHR*) that may play a role during the earliest stages of adventitious root induction [93,94]. In *Arabidopsis* and other plant species, such as rice (*Oryza sativa* L.) or maize (*Zea mays* L.), the establishment of an embryonic root meristem involves members of the GRAS family of putative transcription factors, which include SCARECROW (SCR), SCARECROW-LIKE (SCL), and SHORT-ROOT (SHR) proteins [95,96]. *PrSCL1*, *CsSCL1,* and *PrSHR* were predominantly expressed in roots, root primordia, and in the cambial region of competent cuttings in pine and chestnut [94,97]. Localized and transient increases in mRNA levels were observed in the cambial region and rooting-competent cells, within the initial stages of the adventitious rooting process, before the onset of cell divisions. The expression pattern of *PrSCL1* and *PrSHR* overlapped; nevertheless, *PrSCL1* was induced in the presence of the exogenous auxin needed for cuttings to root, while *PrSHR* induction was not dependent on exogenous auxin [93,94]. The induction of *PrSCL1* in the presence of the exogenous auxin and of *PrSHR* in the absence of exogenous auxin suggests that exogenous auxin-dependent and -independent pathways could be associated with the rooting capacity of tissues. *PrSCL1*, *CsSCL1,* and *PrSHR* expression were also induced in non-competent mature cuttings; however, the mRNA signal was more diffuse and evenly distributed throughout the parenchyma [27,94,97]. Similarly, in black walnut (*Juglans nigra* L.), the highest transcript abundance of *SCARECROW LIKE-1* (*SCL*) gene in rooting-competent cuttings was restricted to root progenitor cells. Tissues not linked to root organogenesis had little change in *SCL* expression. Auxin-treated mature shoots showed an increase in *SHR* and *SCL* expression, but transcript abundance was uniform across all sampled tissues and showed a diffuse mRNA signal [89]. These results indicate that localized transcriptional activation is associated with adventitious root formation in an auxin-, tissue-, and age- or development-dependent manner in Monterey pine, chestnut, and black walnut [27,30,89], suggesting possible conservation mechanisms in the maturation-related decline of AR in distantly related species. Two other genes, *PrSCR* and *PrSCL6*, are expressed in rooting-competent cuttings of Monterey pine during AR but are not detected in non-competent cuttings. While the mRNA level of *PrSCR* is very low in competent cuttings and is not induced by auxin, the expression of *PrSCL6* is induced by auxin in competent cuttings. GRAS genes seem also to be involved in the rooting pathway associated with the enhancement of rooting by diphenylureas in Monterey pine [54,55]. Expression analysis of additional specific members of the pine GRAS family showed that their expression could be involved in complex transcript regulatory networks also associated with auxin, tissue, and age- or developmental-dependent pathways during AR in Monterey pine [27]. These results, along with the identification of three additional *GRAS* genes (two *SCL-3* and one *SCL-7*) expressed in rooting-competent cuttings of chestnut in response to auxin during AR, and the decrease in *SCR2* and *SCL1* gene expression in the stem bases of 36-month-old shoots, showing low capacity of AR, in *Eucalyptus nitens*, reinforce the hypothesis that *SCARECROW-LIKE* genes may play a role during the earliest stages of adventitious rooting in distantly related forest species, and they may act in a redundant or cooperative way controlling gene expression related to the capacity of adventitious rooting at the mature stage [27,30,31,89]. *AUXIN RESPONSE FACTOR (ARF) 6* and *ARF8* expression also increased in rooting-competent parenchyma cells of black walnut in response to exogenous auxin during AR. In addition, *ARF17* expression decreased concomitantly with an increase in *ARF6* and *ARF8* expression. Auxin was necessary for localizing expression changes, as all cell types responded equally and similarly in water-treated cuttings. Again, transcript abundance across all tissues in physiologically mature stems was uniform and showed a diffuse mRNA signal regarding treatment and the time course of AR [89]. Recently, Qi and colleagues described that RETINOBLASTOMA-RELATED proteins in hybrid poplar (*Populus deltoides* × *P. euramericana*) clone “Nanlin 895” play a significant role as a central regulatory node within the *SHR/SCR* network, coordinating both AR development and secondary wall formation, through transcriptional reprogramming of cell cycle regulators and cell wall biosynthesis machinery [98]. In poplar clones showing different rooting ability (high or low), *MYB* and *AP2/ERF* genes were upregulated in the high-rooting group, as well as genes controlling cell wall differentiation [99]. Differential gene expression of other transcription factors involved in the auxin, ABA, ethylene, JA and SA signaling pathways has also been associated with the maturation-related decline of AR in chestnut and oak, according to the potential role of these signaling pathways in the recalcitrance of mature tissues [30,35,38,43]. The differential expression of *MADS-box* genes from the JOINTLESS-like and SHORT VEGETATIVE PHASE groups of transcription factors is remarkable in non-competent microshoots of chestnut [30]. The bulk of ABA signaling could be one of the factors influencing the expression of *MADS-box* genes in mature tissues. Thus, *MADS-box* genes emerge as putative candidates controlling transcriptional responses in mature tissues that may be related to the acquisition of rooting competence in these shoots. In addition, induction of *MADS-box* genes shows a possible function in the integration of different cues, such as ABA and the ontogenetic state, into a gene regulatory network that may regulate the maturation-related decline of AR. In the same way, other genes encoding transcription factors, such as the auxin-responsive *SMALL AUXIN UPREGULATED RNAs (SAURs), GH3-1, AUX/IAA*, the ethylene-responsive genes, the *NAC*, *MYB*, and *MYC2* families, and *AINTEGUMENTA-LIKE*, were also identified as being expressed in mature tissues [30,31]. Therefore, these transcription factors could be related to the capacity of cell reprogramming in mature shoots, resulting in the formation of adventitious roots.

Despite many transcriptomic studies in forest trees, only a small fraction of candidate genes has been functionally validated because tree transformation is slow, regeneration is difficult, rooting is environmentally sensitive, and long generation times complicate knockout analysis. Consequently, true gene-to-rooting proof in trees remains relatively rare, especially at the mature stage of the tree. In hybrid poplar (*Populus tremula* × *Populus alba*) clone INRA 717–1B4, constitutive overexpression of a SHORT-ROOT transcription factor significantly increased adventitious rooting. The root primordium initiation was detected earlier, and the number of adventitious roots was higher. In addition, stronger auxin responsiveness during rooting was detected [100]. On the other hand, knockdown of *PagARF3.1*, a homolog of the *Arabidopsis* Auxin Response Factor 3 in hybrid poplar (*Populus alba* × *Populus glandulosa*) clone cv. ‘84K’, delayed adventitious root formation and reduced root biomass in transgenic plants, whereas overexpression of *PagARF3.1* promoted earlier rooting and increased the number of adventitious roots. Results indicate that *PagARF3.1* acts as a positive regulator of adventitious root formation by repressing IPT-mediated cytokinin biosynthesis [101]. In birch (*Betula pendula* Roth) and hybrid walnut “Zhongningsheng” (*J. hindsii* × *J. regia*, “ZNS”), overexpression of *WOX11-LIKE* genes promoted early root initiation and significantly increased ARs [102,103]. This research was conducted using competent cuttings for rooting; therefore, it does not provide data on whether these genes play a role in the reprogramming of non-competent adult cells.

The limitation of the reprogramming potential could be related to the presence of signals in tissues that retain a physiological or developmental memory. The chromatin status and epigenetic mechanisms that give rise to specific nuclear architecture may play a role in controlling cellular plasticity [104]. Early studies on the epigenetic regulation of the transition from the juvenile to the adult phase revealed differences in the degree of DNA methylation [105]. In chestnut, expression of genes encoding proteins involved in DNA methylation and small interfering RNA, or histone demethylation (JMJ16), deacetylation (SRT1), and binding (VIN-3-like protein 2) was detected in rooting-competent juvenile microshoots under AR conditions. Genes that are differentially expressed by non-competent adult microshoots encode proteins involved in DNA and histone methylation, Argonaute-1-mediated degradation of miRNA-targeted RNAs, and RNA-directed DNA methylation [30]. The expression of specific histone deacetylases (*SRT1*) in juvenile tissues could be related to the effect of ethylene on AR. In *Arabidopsis*, *AtSRT1* has been shown to repress the expression of several ethylene-responsive genes via histone acetylation [106]. Therefore, the inhibitory effect of ethylene on adventitious rooting in adult shoots of chestnut that cannot root—an effect not observed in juvenile shoots that can root—could be associated with an epigenetic-based mechanism in rooting-competent juvenile shoots.

A shift in microRNA (miRNA) expression profiles has also been described [107,108,109,110]. Conserved miRNAs differentially expressed between juvenile, adult, and rejuvenated adult *Ilex paraguariensis* A. St.-Hil. plants were identified. The expression of miR156 gradually decreased as the plant transitioned from juvenile to adult stages. In contrast, miR172 was predominantly expressed in adult plants. The analysis of miRNA transcriptomic profiles between juvenile, adult, and rejuvenated adult plants by serial cuttings confirmed a decrease in miR172 associated with the progression of serial propagation, and an increase in miR156 expression related to the rejuvenation of adult plants. This variation in the miRNA profiles was correlated with the expression of genes encoding components of the sugar signaling and transcription factors of the GRAS family, such as SCR, SHR, and SCL, indicating its possible role in the reversion to a juvenile state that could be related to the reprogramming potential and promotion of AR [107]. Xu and colleagues provided insights into the mechanism by which a miRNA-directed signaling pathway dynamically coordinates mitochondrial functionality with adventitious rooting capacity in *Populus tomentosa* via PAT and homeostasis that contributes to the formation of auxin maxima at rooting-competent cells [108]. miR476a, a member of the MIR476 family, functions as a novel regulator of wound-induced AR formation. MiR476a directly targets several mitochondrion-localized PPR genes belonging to the restorer-of-fertility-like (RFL) gene family during AR. RFL encodes mitochondrion-localized pentatricopeptide repeat (PPR) proteins, thereby restricting mitochondrial energy production that is unfavorable to AR. MiR476a-dependent suppression of RFLs leads to the enhancement of the mitochondrial status to increase the energy supply. Mitochondrial retrograde signals activate the expression of the auxin transport genes *PIN-FORMED2/5b* (*PIN2/5b*), which modulate the polar transport and homeostasis of auxin in rooting-competent cells and promote AR formation by increasing the expression of key AR-associated genes. These findings suggest that mitochondria-directed energy homeostasis may play a key role in AR formation. In hybrid poplar (*Populus deltoides* × *Populus euramericana*) cv. ‘Nanlin895’, miR159a also plays a positive role in regulating the development of AR by inhibiting *PeMYB33* expression in a post-transcriptional manner and reducing the sensitivity to ABA [109]. In addition, long non-coding RNAs have also been involved in adventitious rooting. In *Populus deltoides* × *P. euramericana* cv. ‘Nanlin 895’, lncWOX11a acts as a negative regulator of adventitious rooting by downregulating the *WUSCHEL*-related homeobox gene *WOX11*, which is related to the induction of adventitious root development [110]. The differential expression of several epigenetics-related genes associated with AR and with the maturation-related decline of AR suggests that morphogenic responses are under epigenetic control, integrating several signals, such as maturation, phytohormone signaling, or transcriptional expression networks.

## 6. Conclusions

**Figure 1:**
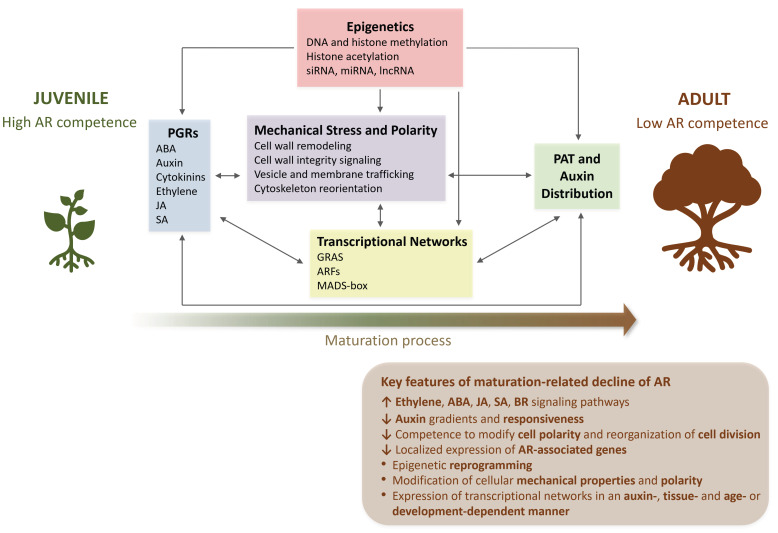
Maturation-related decline in adventitious rooting formation (AR) in forest tree species. ABA—abscisic acid, JA—jasmonic acid, PAT—polar auxin transport, SA—salicylic acid.

Age- and maturation-related trends result from complex interactions among extrinsic and intrinsic factors. Integration of the function of long-distance regulators with cellular signals or transcriptional networks, including epigenetic factors, in rooting-competent cells and/or developing or differentiating cells following unique developing pathways, is a major future challenge to understand recalcitrance. Furthermore, cell dynamics mediated by physical, chemical, and mechanical properties of the cell will provide a complete understanding of maturation-related decline of AR in forest tree species (Figure 1). Increasing evidence suggests the existence of extranuclear auxin signaling pathways that cut short auxin-induced changes in nuclear gene expression. These new pathways rely on auxin receptors localized outside the nucleus and/or on transmembrane kinase receptors localized in the plasma membrane [111]. Furthermore, comparisons of the different signaling pathways among different forest species, preferably those that are phylogenetically distant, will help identify potential conserved mechanisms that could pave the way for practical applications aimed at improving AR in adult plants. Molecular analysis of the mechanisms underlying the maturation-related decline of AR, combined with cutting-edge technologies for analyzing multigene expression profiles, will enable the identification of an expression signature that characterizes the specific levels of regulation and the factors involved in the recalcitrance for AR at the mature stage of development. This expression signature could be used to predict recalcitrance and could provide additional tools for its modification.

In this regard, rejuvenation and manipulating cellular plasticity are key practical strategies to overcome the decline of AR in mature forest trees. Success should be achieved by combining hormonal treatments, modification of epigenetic signatures, and targeted cutting propagation to reset adult cells into a competent state for root regeneration. Applying chemical modulators of gene expression networks may restore developmental competence in recalcitrant adult tissues by increasing cellular flexibility, allowing direct reprogramming into root primordia. Gene edition and GMO technologies may bypass this bottleneck by modifying gene expression networks or balancing hormonal ratios, and reprogramming somatic cells back into a juvenile, root-competent state. Transcription factor modulation, hormone pathway engineering, cytoskeleton and cell-wall remodeling for making adult tissues responsive to root induction treatments, or epigenetic reprogramming may help “reset” the physiological age of explants, retaining juvenile traits and restoring their capacity to regenerate adventitious roots. This may allow us to minimize the maturation-related decline that typically begins before or as trees transition to reproductive phases. Integrating these tools, ranging from nursery methods that can be implemented immediately to advanced molecular approaches, can make large-scale clonal forestry from mature, elite trees viable.

## Data Availability

No new data were created or analyzed in this study. Data sharing is not applicable to this article.
